# A CFD study on the interplay of torsion and vortex guidance by the mitral valve on the left ventricular wash-out making use of overset meshes (Chimera technique)

**DOI:** 10.3389/fmedt.2022.1018058

**Published:** 2022-12-22

**Authors:** Federico Canè, Lucas Delcour, Alberto Cesare Luigi Redaelli, Patrick Segers, Joris Degroote

**Affiliations:** ^1^ IBiTech – bioMMeda, Department of Electronics and Information Systems, Ghent University, Ghent, Belgium; ^2^ Department of Electromechanical, Systems and Metal Engineering, Ghent University, Ghent, Belgium; ^3^ Department of Electronics, Information and Bioengineering, Politecnico di Milano, Milano, Italy

**Keywords:** left ventricle, ventricular torsion, mitral valve, Chimera technique, overset meshes, residence time

## Abstract

Cardiovascular disease often occurs with silent and gradual alterations of cardiac blood flow that can lead to the onset of chronic pathological conditions. Image-based patient-specific Computational Fluid Dynamics (CFD) models allow for an extensive quantification of the flow field beyond the direct capabilities of medical imaging techniques that could support the clinicians in the early diagnosis, follow-up, and treatment planning of patients. Nonetheless, the large and impulsive kinematics of the left ventricle (LV) and the mitral valve (MV) pose relevant modeling challenges. Arbitrary Lagrangian-Eulerian (ALE) based computational fluid dynamics (CFD) methods struggle with the complex 3D mesh handling of rapidly moving valve leaflets within the left ventricle (LV). We, therefore, developed a Chimera-based (overset meshing) method to build a patient-specific 3D CFD model of the beating LV which includes a patient-inspired kinematic model of the mitral valve (LVMV). Simulations were performed with and without torsion. In addition, to evaluate how the intracardiac LV flow is impacted by the MV leaflet kinematics, a third version of the model without the MV was generated (LV with torsion). For all model versions, six cardiac cycles were simulated. All simulations demonstrated cycle-to-cycle variations that persisted after six cycles but were albeit marginal in terms of the magnitude of standard deviation of velocity and vorticity which may be related to the dissipative nature of the numerical scheme used. The MV was found to have a crucial role in the development of the intraventricular flow by enhancing the direct flow, the apical washout, and the propagation of the inlet jet towards the apical region. Consequently, the MV is an essential feature in the patient-specific CFD modeling of the LV. The impact of torsion was marginal on velocity, vorticity, wall shear stress, and energy loss, whereas it resulted to be significant in the evaluation of particle residence times. Therefore, including torsion could be considered in patient-specific CFD models of the LV, particularly when aiming to study stasis and residence time. We conclude that, despite some technical limitations encountered, the Chimera technique is a promising alternative for ALE methods for 3D CFD models of the heart that include the motion of valve leaflets.

## Introduction

Cardiovascular disease is the main cause of death worldwide, accounting for 17.8 million deaths expected to increase up to 23.6 million by 2030 (1). Most often, this entails a chronic process whereby pathophysiological changes in cardiac function progress slowly (e.g., development of cardiac hypertrophy, cardiac dilatation, valvular dysfunction) and unnoticed, until reaching a point of no return with the development of symptoms, or occurrence of a fatality as myocardial infarction or stroke. In many cardiovascular pathologies, unphysiological intraventricular hemodynamics is a consequence and/or direct cause of the pathology (e.g., valve dysfunction), leading to flow disturbing and energy dissipating jets, altered intraventricular swirling patterns with less energy-efficient filling and emptying of the ventricular chamber, and effects on blood residence times within the cardiac cavity. Furthermore, therapeutic interventions - especially those related to resting valve function - may drastically alter flow patterns within the heart cavities. A fundamental understanding of intraventricular hemodynamics should lead to an improved assessment of deviations from normal physiological flow, earlier detection of chronic abnormalities, better assessment of therapeutic efficacy, and further refinements in surgical techniques or cardiovascular medical device design.

Among the techniques used to investigate the intraventricular flow field, Computational Fluid Dynamics (CFD) modelling offers the advantage to compute flow metrics with finer spatial and temporal resolution than possible with any available *in vivo* imaging technique. Compared to in-vitro benchmarks, it is much more flexible in performing parametric and comparative studies thanks to the ease of tuning and isolating the parameter under investigation. Therefore, with the relentless improvement of computational power and the growth of computational models, it would be paramount if patient-specific CFD simulations could finally be used as a clinical support tool in cardiovascular surgical planning and diagnostics (2).

The investigation of the intraventricular flow features in relation to the LV pathophysiological condition has been evaluated with several flow-based biomarkers. The diastolic vortex formation interplays both with the pumping efficiency (such as flow washout, ventricular energetics) and the biological nature of the blood flow (blood clots). This dual-side efficiency has been thoroughly investigated focusing on sustaining the blood motility up to the apex and, preventing thrombus formation, and has become a key feature in terms of wash-out of the ventricular chamber and ventricular energetics (3). The clinical contribution of CFD modelling is potentially multidisciplinary and groundbreaking, ranging from the early detection of cardiovascular diseases (heart failure conditions, pulmonary hypertension, aortic and cerebral aneurysms, aortic dissection, cardiac valve pathologies), to the assessment of cardiovascular devices and therapies (prosthetic valves, stent, and ventricular assist device placement, cardiac resynchronization therapy) (4). The MV leaflets motion can be computationally replicated *via* a kinematic model, preferably based on measurements from 4D medical images, or a Fluid-Structure Interaction (FSI) model (5).

The first MV models were highly simplified, implementing the valve as a planar orifice that was either open or closed [on-off approach (6–8)] or with a time-varying cross-sectional area (6, 9, 10), therefore neglecting the MV leaflets' configuration, their kinematics and interaction with the flow field. Next generation models considered both the morphology and the motion of the leaflets. These valve models can be classified into the patient-inspired (often based on parametric models) or patient-specific MV models, segmented from medical images. Among the patient-inspired MV models: (i) Chnafa et al. (11–13) segmented the moving MV annulus from 4D CT scans and modeled the MV leaflets as a continuous elliptical shape with a given thickness, which instantaneously switches between the open and the closed configuration, thus neglecting the opening and closing phases; (ii) Seo et al. (14) defined the MV morphology based on the anatomical measurements by Ranganathan et al. (15) with the Anterior (AL) and Posterior (PL) leaflets following rigid rotations with different angular profiles. Contributions among patient-specific MV models are: (i) Mihalef et al. (16) modeled the whole heart using machine-learning algorithms to robustly estimate the patient's morphological and functional parameters from multiple 4D CT datasets with claimed precision of 90%; (ii) Bavo et al. (17) segmented the LV and MV kinematics from 4D transesophageal echocardiographic images.

Even though patient-specific MV models based on 4D medical images can account for the fluctuations and curvature changes, the limited thickness of the leaflets (around 2 mm), compared to their other dimensions, in combination with their fast opening and closing dynamics, makes it challenging for the currently used imaging modalities to visualize the valve with a sufficiently high spatial and temporal resolution, especially during the opening and closing phases. A whole branch of research is dedicated to combining the different imaging techniques to enhance their advantages. In addition to the 4D medical images used to derive geometrical and kinematical patient-specific models (ultrasound, CT or MRI), the 4D flow MRI is a promising cardiac imaging technique, which allows to quantify the intracardiac flow without the need of performing CFD modelling. Nonetheless, at the current stage, it still suffers from a coarser spatial and temporal resolution in comparison with CFD and FSI simulations (18). In this case, the motion of the ventricular walls is entirely prescribed by medical images, with 1 temporal configuration the mitral valve segmented by medical images, and thus not computed from the resulting pressures. Hence, the model is Computational Fluid Dynamics (CFD) with moving boundary but not Fluid-Structure Interaction (FSI)’.

On the other hand, FSI modeling requires: (i) the definition of the constitutive and structural properties, which is more straightforward for materials composing prosthetic mechanical valves rather than the natural leaflets; (ii) a finer description of the MV anatomy including the details, such as the chordae tendinae and papillary muscles, not essential in kinematic models. For these reasons, patient-specific FSI models coupling the LV and MV rarely succeeded to combine simultaneously multiple features: the patient-specific LV model with both mitral and aortic valves by Su et al. (19) is limited to a 2D geometry, otherwise the 3D MV is placed within a fixed tube (18) or limited to the filling phase (20). Recently, there have been a few breakthroughs in coupling patient-specific MV and LV models with FSI and they have been used to study the effects of impaired myocardial active relaxation (21) or the transapical neo-chordae implantation (22, 23).

Regardless of whether the MV kinematics is prescribed in CFD simulations or calculated in FSI simulations, the computational approaches typically used to solve the flow (and structural) equations of problems involving moving meshes can be boundary-conforming, such as the Arbitrary Lagrangian-Eulerian (ALE) approach, or non-boundary conforming, such as the Immersed Boundary Method (IBM). In the former, the boundary mesh deforms accordingly to the motion of valves in providing an accurate WSS computation on their surface. In the latter, the effect of the moving immersed body on the fluid is taken into account by adding a source term in the Navier–Stokes equations resulting in a more suitable approach for complex geometries. In addition to these most common techniques, one has to mention: the smoothed particles hydrodynamics, which is a meshless approach used successfully to overcome the lack of complete valve coaptation during systole (24, 25); the Chimera (or overset) technique, which is particularly suitable in handling problems with different components in motion, used to investigate the hemodynamics within a pulsatile left ventricular assist device (26). The Chimera technique has the advantage over ALE that it allows tackling the separation of the fluid domain that occurs during the valve coaptation without a local fictitious increase of viscosity or added source term in the Navier-Stokes equations, whereas, over the IBM approach, complex geometries can be represented with boundary layer meshes resulting in a more accurate computation of interface wall shear stress.

In previous work, we built a patient-specific CFD model of the LV chamber based on the Chimera technique. Firstly, we generated a semi-automatic algorithm to generate 4D meshes of the LV with 1-to-1 vertex correspondence to replicate the patient-specific cardiac motion (27). Secondly, we investigated the impact of the LV torsion on the fluid dynamics using the Chimera technique (28). A major limitation of that study, however, was the absence of the mitral valve in the model, known to highly impact the intraventricular flow field and a key structural element in the formation of the ventricle-filling vortex ring in diastole. Therefore, we propose a workflow to build patient-specific CFD models of the LV with a kinematic model of the MV based on the Chimera technique, which is promising to tackle the several challenges involved in cardiac modeling. Bearing in mind that many cardiac pathologies (cardiomyopathies, hypertrophy, diabetes, hypertension, ischemia, normal aging) influence ventricular torsion, simulations are performed in a model with and without physiological torsion.

## Materials and methods

### Medical imaging dataset segmentation

The heart of a 27 years old healthy male volunteer was scanned using a cine-MRI short-axis dataset and a cine-MRI radial dataset at the Policlinico San Donato using a Gradient Recalled Echo (GRE) sequence within 30 time-instants spanning 1 cardiac cycle. The study was performed with the ethical approval of the hospital and the informed consent of the subject. The short axis dataset had an in-plane resolution of 1.17 mm and a through-plane resolution of 8 mm, whereas the radial dataset shared the same in-plane resolution and was acquired every 10° along the rotation axis passing through the LV apex and MV centroid. The 4D geometries of the LV were manually segmented using Materialise Mimics 18.0® from the cine-MRI short-axis dataset, while the 4D annulus and one configuration of the leaflets at the A-wave peak, due to high uncertainty linked with the highly impulsive leaflets and the low spatial and temporal resolution, were segmented from the cine-MRI radial dataset within an in-house Matlab-code developed by the Biomechs groups of Politecnico di Milano.

### Mesh generation

The Chimera (or overset) technique defines the shape of the fluid domain with one or multiple component Boundary Layer (BL) grids attached to the moving walls that are overlapping a 3D Cartesian grid which is stationary. Interpolation schemes transfer the flow field solution (pressure and velocities) from the component to the background mesh, and vice versa. The Chimera technique is particularly helpful when dealing with complex deforming geometries, and avoids complex remeshing of an ALE technique. A detailed description of the Chimera technique can be found in the Methods section of Canè et al. (29).

**Figure 1:**
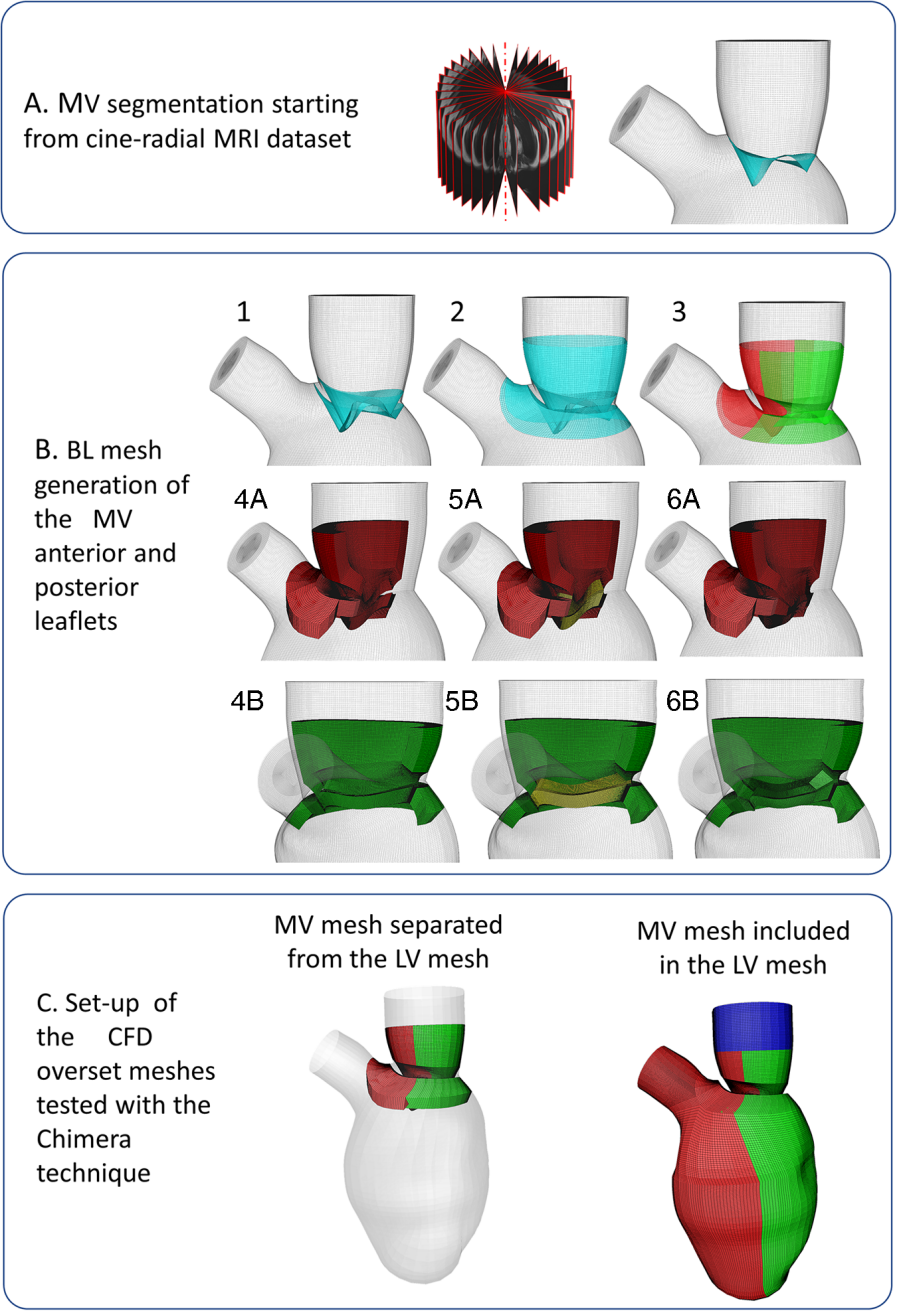
(panel **A**) Mitral valve segmentation from cine-radial MRI images; (panel **B**) Creation of the mitral valve mesh; (panel **C**) Tested meshes configurations: not-working separated LV-MV configuration on the left, working conformally connected fused LV-MV on the right.

In this section, we describe the steps taken to generate three component grids: (i) one anterior MV mesh connected conformally to the anterior part of the LV mesh; (ii) one posterior MV mesh connected conformally to the posterior part of the LV mesh (posterior LVMV mesh, shown in green) and (iii) an inlet plug mesh overlapping with both LVMV meshes to close the fluid domain (Figure 1C: conformal fused LVMV).

The surface mesh of the MV leaflets is generated by connecting the MV annulus, modeled in a static configuration defined from the averaging of its 4D configurations over a cardiac cycle, with the profile of the MV free edge of the valve at the A-wave peak (Figure 1A). The anterior and posterior BL MV meshes were created around the MV surface, bearing in mind that they represent the fluid region in contact with the MV surface mesh, following these steps:

(1) Two copies of the segmented MV surface mesh (represented in cyan) were generated and translated, sliding along the LV endocardial mesh, so that the space encompassed between the two copies (shown with the dotted red line in Figure 1.B.1) represents the MV with a thickness of 2 mm. Subsequently, the lower edges of the copies are connected (shown with the yellow line, Figure 1B.1);
(2) A collar mesh (shown with a black line in Figure 1B.2) overlapping with the LV endocardial surface mesh at the upper and lower part of the leaflets is created and fused with the surface leaflet meshes, generated in the previous step;
(3) The resulting mesh is divided into the anterior and posterior leaflets (shown in red and green, respectively, in Figure 1B.3), sharing six overlapping layers, with the following steps performed both for the anterior and posterior leaflets;
(4) As preparation to build the three-blocks BL mesh for each leaflet, the surface mesh of this region is divided into three surfaces: the upper mesh, which is composed of the upper collar and upper leaflet grids (shown in orange, Figures 1B.4A,B), the free edge (shown in blue, Figures 1B.4A,B), the lower mesh (composed of the lower collar and lower leaflet, shown in magenta, Figures 1B.4A,B). Separately for the anterior and posterior leaflet (Figures 1B.4A,B), the upper and lower surface meshes are extruded with a thickness of 4 mm to build the first two blocks of each leaflet (shown in red and green, in Figures 1B.4A,B);
(5) The third block is generated by extruding the surface shown in yellow (Figures 1B.5A,B), resulting from the fusion between the free edge surface and the contiguous surfaces created at step 5, towards the inner direction with a thickness of 4 mm (Figures 1B.6A,B).

**Figure 2:**
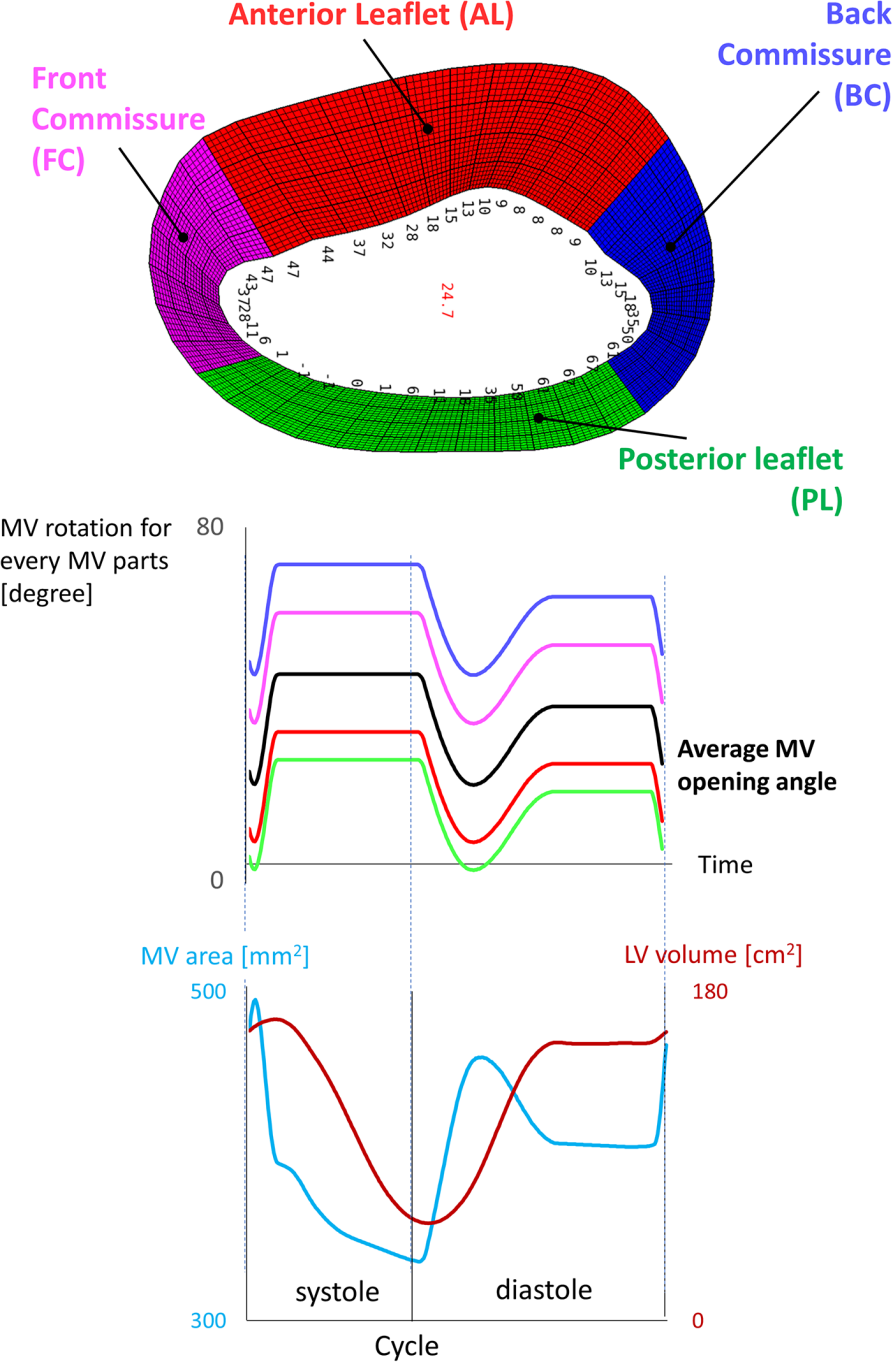
Kinematic model of the mitral valve reporting the average angle for each zone of the valve.

The mesh separation between the anterior and posterior leaflet (step 3) is fundamental during valve closure because the Chimera technique allows the intersection between different component grids, but not self-intersection, which may become problematic during valve closure.

We enabled manual cut-control and ensured that the overset interface occurred along a vertical direction between the anterior and posterior LVMV meshes wall and along a horizontal direction between the inlet plug and the anterior and posterior LVMV meshes. The overlapping between the component grids was conformal on the external surface of the three boundary layer meshes. The final three component grids are displayed in Figure 1C (anterior LVMV mesh in red; posterior LVMV mesh in green and an inlet plug mesh overlapping with both LVMV meshes to close the fluid domain in blue).

The opening angles were not assigned homogeneously to the leaflets, but *via* a shaping function that divided the MV into four zones (anterior and posterior leaflet, front and posterior commissure) (Figure 2). The finer control was essential to leave more space during early diastole between the MV leaflets and the LV wall in correspondence with the commissures, which otherwise would have been too narrow to fit the valve, resulting in negative volume errors. The quality criteria used for the 4D generated meshes are a maximum skewness of 0.9 and a minimum scaled Jacobian of 0.02. The number of mesh elements is 737 k, 496 k, 346 k, 2.5 M for the anterior LVMV grid, the posterior LVMV grid, the inlet plug, and the background grid, respectively.

The mesh sensitivity analysis was performed in our previous study without the mitral valve (28), evaluating the combinations of a background grid with an edge length of 0.5 mm and 0.7 mm and a component grid with 100 k and 300 k elements. The comparison of the different meshes was based on the investigation of the WSS, the endocardial pressure, pressure, and velocity along predefined trajectories. A finer mesh resolution of the component boundary layer grid allows for a finer evaluation of the flow velocity profile and hence the wall shear stress without significant impact on the computed variables or the computational time. Therefore, the component grid with 300 k elements was chosen, in combination with the background grid with an edge length of 0.5 mm. In the case with the MV, the near-wall mesh resolution was refined to increase the overlapping between the component grids, resulting in 737 k, 496 k, and 346 k for the anterior LVMV grid, the posterior LVMV grid, the inlet plug, respectively. A background grid with an edge length of 0.7 mm (2.5 M elements) was chosen to solve an unexpected vanishing error of the fluid zone corresponding to the posterior LVMV grid when using a background grid with an edge length of 0.5 mm.

### Torsional implementation

Torsion has been applied as a rotation of the LV sac with respect to the vertical axis passing through the MV centroid and the apex. The intensity of the rotation varies linearly during the cardiac cycle and is defined with a piecewise ramp-like function: the twist and the uncoil occur during systole and diastole, respectively. The maximum rotational angle occurs at end-systole and, in the physiological torsion (referred to as Torsion) case, it was based on the measurement of the angle encompassed by the centroid of the papillary muscles between end-diastole and end-systole (*γ* = 13°). In a second simulation, torsion was omitted (referred to as No Torsion).

### Temporal interpolation

The anterior and posterior LVMV meshes were generated on the existing and already interpolated 4D BL meshes of the LV using a Natural Cubic spline, resulting in 290 temporal configurations spanning one cardiac cycle and a time step of 3 ms. For more details on the temporal interpolation method, the reader is referred to (28).

### CFD set-up

Our Chimera-based CFD model was defined in Fluent 2019 R3®, with the fluid domain represented by three BL component grids (anterior LVMV, posterior LVMV, inlet plug) embedded in a 3D Cartesian grid. The initialization of the overset interface subdivided the cells of the meshes into four categories (dead, solve, receptor, donor) and established the connectivity between the participating zones to exchange variables during the computation of the Navier-Stokes equations. The cut-control feature was required for finer control on the cells to disable (dead cells), allowing to specify the wall zones not to be cut by the fluid zones. In our case, the wall zones of the anterior and posterior LVMV meshes were excluded from the cutting of the posterior and anterior fluid zones, respectively.

As in (28), a coupled solver scheme was used for the pressure-velocity coupling, together with a 2nd order upwind scheme for the convective terms and a 1st order implicit scheme for the time discretization. Blood was modeled as a homogeneous and Newtonian fluid [*ρ *= 1,060 kg/m^3^, *μ *= 0.003 kg/(m·s)]. Alternating on-off boundary conditions were imposed as follows: inlet pressure set at 7 mmHg and outlet as a wall during diastole; outlet pressure set at 120 mmHg and inlet as a wall during systole. Our model is without turbulence model. The flow regime inside the LV is debatable due to the highly transient dynamicity involved and, in agreement with Chnafa et al. (11–13), we believe that the main large-scale hemodynamic features (such as jets, main vortices, and ejection) can be characterized even with the laminar flow assumption. Six cardiac cycles were simulated on a Dell PowerEdge R620 server (2 × Intel Xeon E5-2680v2 CPUs at 2.8 GHz) and the HPC-UGent cluster system. In the former, one cardiac cycle was computed in about 24 h using eight cores. In the latter, the computation time depended on the number of nodes and the architecture of the available cluster unit.

### Post-processing

The comparison of the fluid dynamics in the simulated cases is based on the computation of the velocity and vorticity magnitude, the energy loss (EL), the wall shear stress (WSS) distribution, and the Residence Time (RT). The velocity and vorticity are separated into classes (reported in Supplementary Material Tables S1–S6 in the Supplementary Material) in ascending order according to the magnitude to quantitively assess the differences of the contours and are evaluated in three short-axis planes (SA1, SA2, SA3, from top to bottom) and one long axis plane (LA).

The EL is computed from the viscous term of the incompressible Navier-Stokes, as follows:

$$\Phi_{V}=\frac{1}{2}\mu \overset{3}{\underset{i=1}{\sum }}\overset{3}{\underset{\;j=1}{\sum }}{\left[\left(\frac{\partial u_{i}}{\partial x_{j}}+\frac{\partial u_{j}}{\partial x_{i}}\right)\right]}^{2}$$

$$E_{L}=\int_{t0}^{t1}\overset{\mathrm{num}\;\mathrm{voxels}\;}{\underset{i=1}{\sum }}\phi_{V}V_{i}\;dt$$

where $\frac{1}{2}\left[\left(\frac{\partial u_{i}}{\partial x_{j}}+\frac{\partial u_{j}}{\partial x_{i}}\right)\right]$ is the strain rate tensor, *V*_*i*_ the volume of the cell (0.343 mm^3^), µ the blood viscosity [0.003 kg/(m·s)], and dt the time step of the integration.

Regarding the RT, 4,500 massless particles were seeded at the nodes of the inlet surface mesh at the beginning of the 5th diastole and moved along the velocity field during the last two cardiac cycles, using the particle paths computation in Tecplot 360 EX 2,019 R1. For the RT computation, the particles were classified into three categories: (i) ejected within the 1st beat (direct flow); (ii) ejected within the 2nd beat; (iii) residing in the LV after 2 beats.

Both the EL and the RT were evaluated during the 5th and 6th cardiac cycles, whereas the remaining variables during characteristic time-points of the 6th cardiac cycle. The chosen characteristic time-points were the diastolic E-peak and A-peak and end-systole.

The cycle-to-cycle variation was estimated by computing the median, 5th and 95th percentile of velocity and vorticity magnitude, pressure, and velocity components, within three spherical cloud points (N_points_ = 1,000 points, radius = 5 mm) distributed along the axis passing through the MV centroid and the LV apex. In addition to studying the impact of torsion, we also compared results to a case without the presence of the MV. We also assessed the impact of the cycle-to-cycle variation by computing the standard deviation of the velocity and vorticity magnitude in the LA plane at the diastolic E-peak, being the time step with the largest velocity gradient. The reported quantities were computed using data from the 3rd–6th cycle and evaluated for the three simulated cases (LV Torsion, LVMV No Torsion, LVMV Torsion).

In the bulls' eye representation, which divides the LV endocardium into 17 sectors, we reported the mean and maximum WSS as the difference in every sector between the investigated cases (Torsion - No Torsion, LVMV - LV) to highlight visually the zones impacted by Torsion and the MV. Visually, the first case (Torsion, LVMV) dominates in the red zones, whereas the second case (Torsion, LV) in the blue zones.

## Results

### Influence of torsion on intraventricular hemodynamics

**Figure 3:**
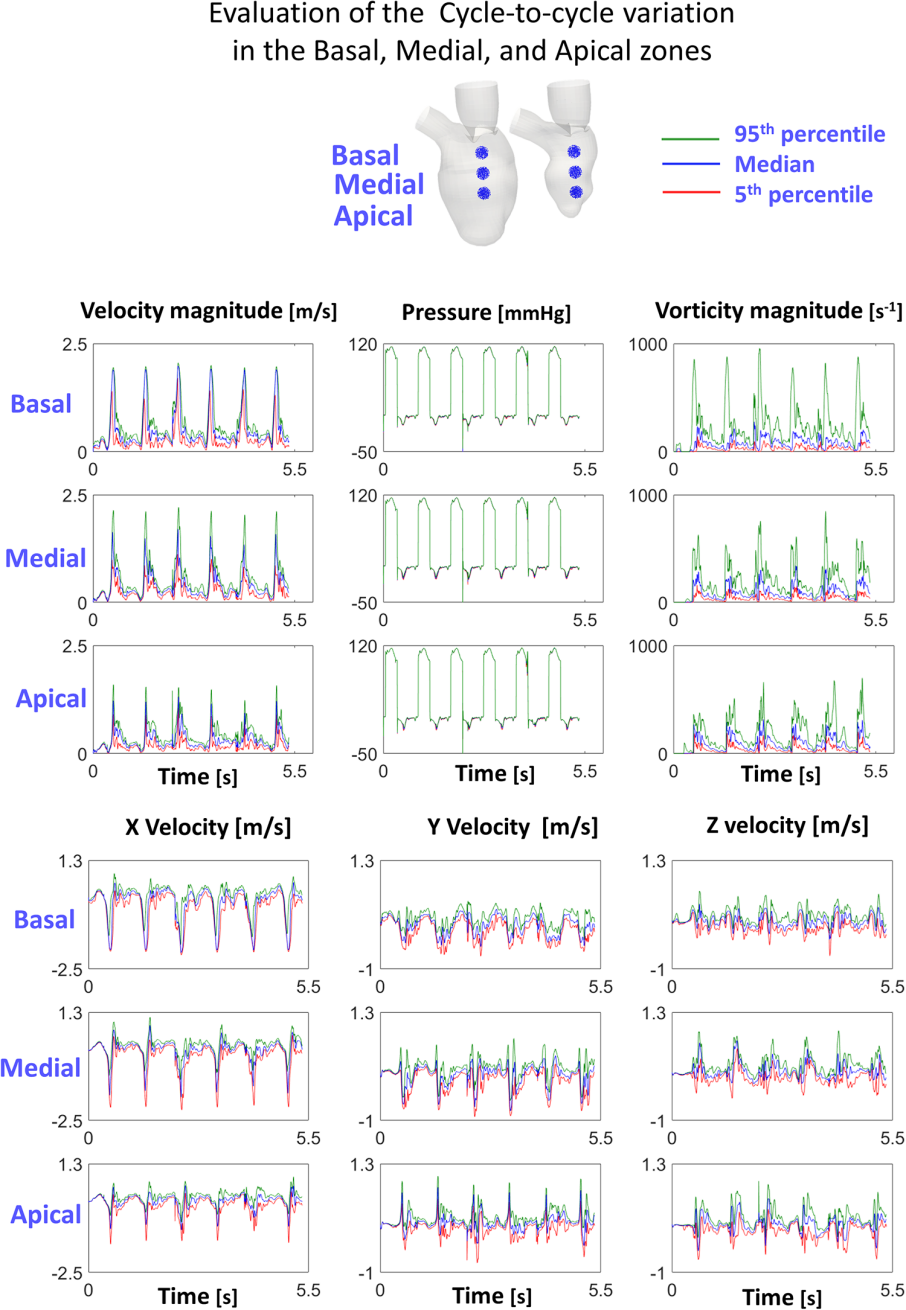
Cycle-to-cycle variation in the investigated cloud points shows that the transitional effects are still relevant.

Figure 3 displays 5th percentile, median, and 95th percentile values of velocity (magnitude and components), pressure, and vorticity as a function of time within the three control volumes for the reference case with physiological torsion and the presence of the mitral valve. Cycle-to-cycle variation is still prominent after six cardiac cycles, especially for vorticity and y and z components of velocity. The evaluation of the standard deviation of the velocity and vorticity magnitude allowed us to quantify the impact of the cycle-to-cycle variation. Even though the cycle-to-cycle variation is still visible after six cycles, the maximum value of the standard deviation at the E-peak is 3.8 10^−8^ m/s and 3.5 10^−5^ s^−1^ in the LA plane at the diastolic E-peak for the velocity and vorticity magnitude, respectively (Figure 6). The higher values of the standard deviation are reached within the tract between the atrium and ventricle, and their magnitude is amplified by the presence of the MV.

**Figure 4:**
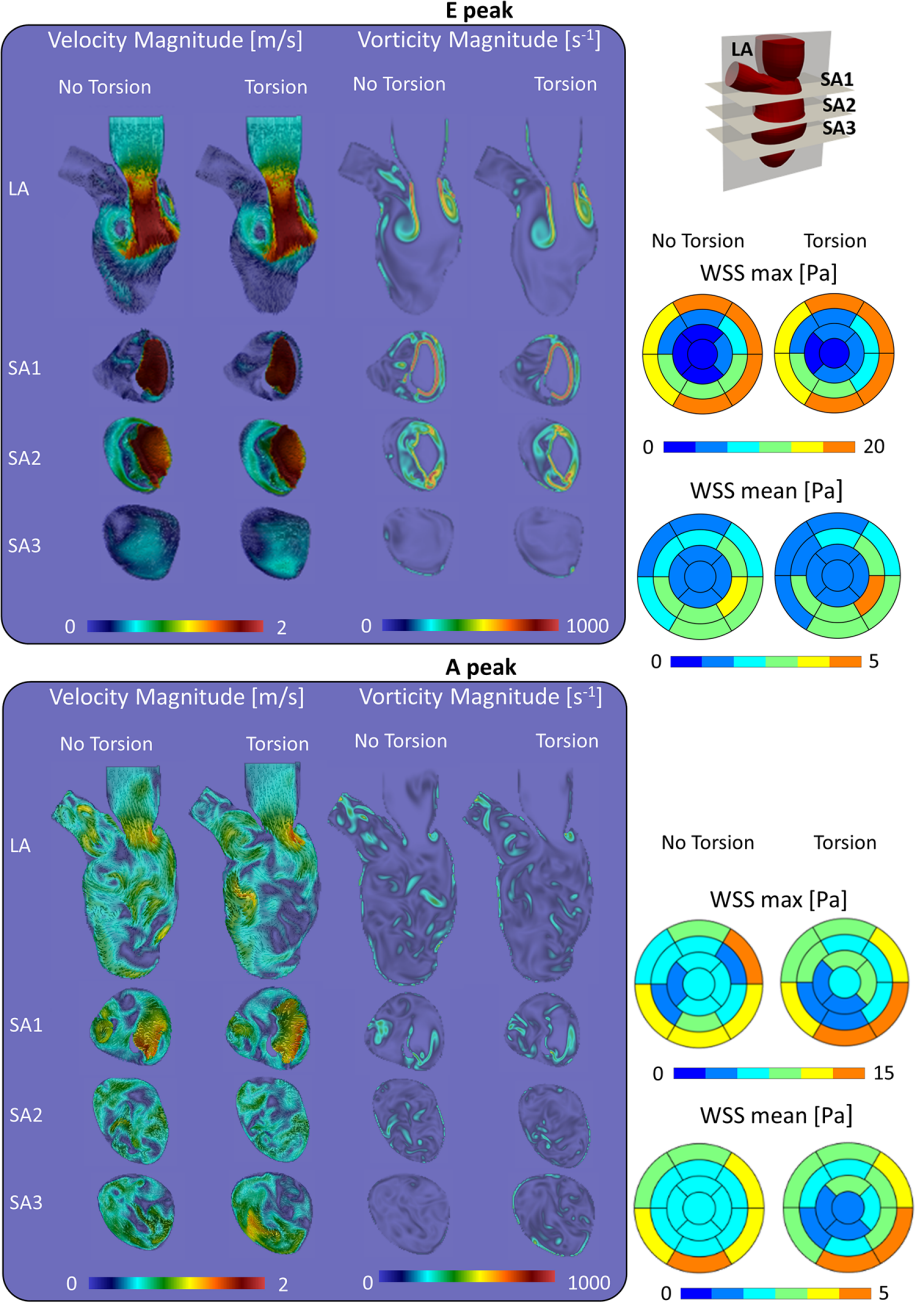
Velocity, vorticity and wall shear stress during the diastolic peaks show a marginal impact of torsion on the investigated variables.

At the E-peak of diastole (Figure 4, upper panel), the inlet jet reaches the peak velocity magnitude of 2.0 m/s with the formation of two vortical structures that impinge the posterior endocardial wall in every simulated case. Both the velocity and vorticity distributions are highly comparable between the Torsion and No torsion cases (Supplementary Material Tables S1,S2). Omitting torsion leads to a slight increase in velocity magnitude in the long axis (LA) and SA1 planes and a mild decrease in the SA3 plane; a slight increase in vorticity is found in the SA1 plane.

At the diastolic A-peak (Figure 4, bottom panel), two main features can be noted regardless of the torsional degree: (i) both the peak of velocity (0.7 m/s) and vorticity (800 s^−1^) lost intensity; (ii) the two main E-peak vortex structures dissipated into smaller vortices more uniformly distributed in the LV domain. Disregarding torsion induces a mild increase in velocity magnitude in the LA, SA1 and a moderate increase in the SA3 plane (Supplementary Material Tables S3,S4).

**Figure 5:**
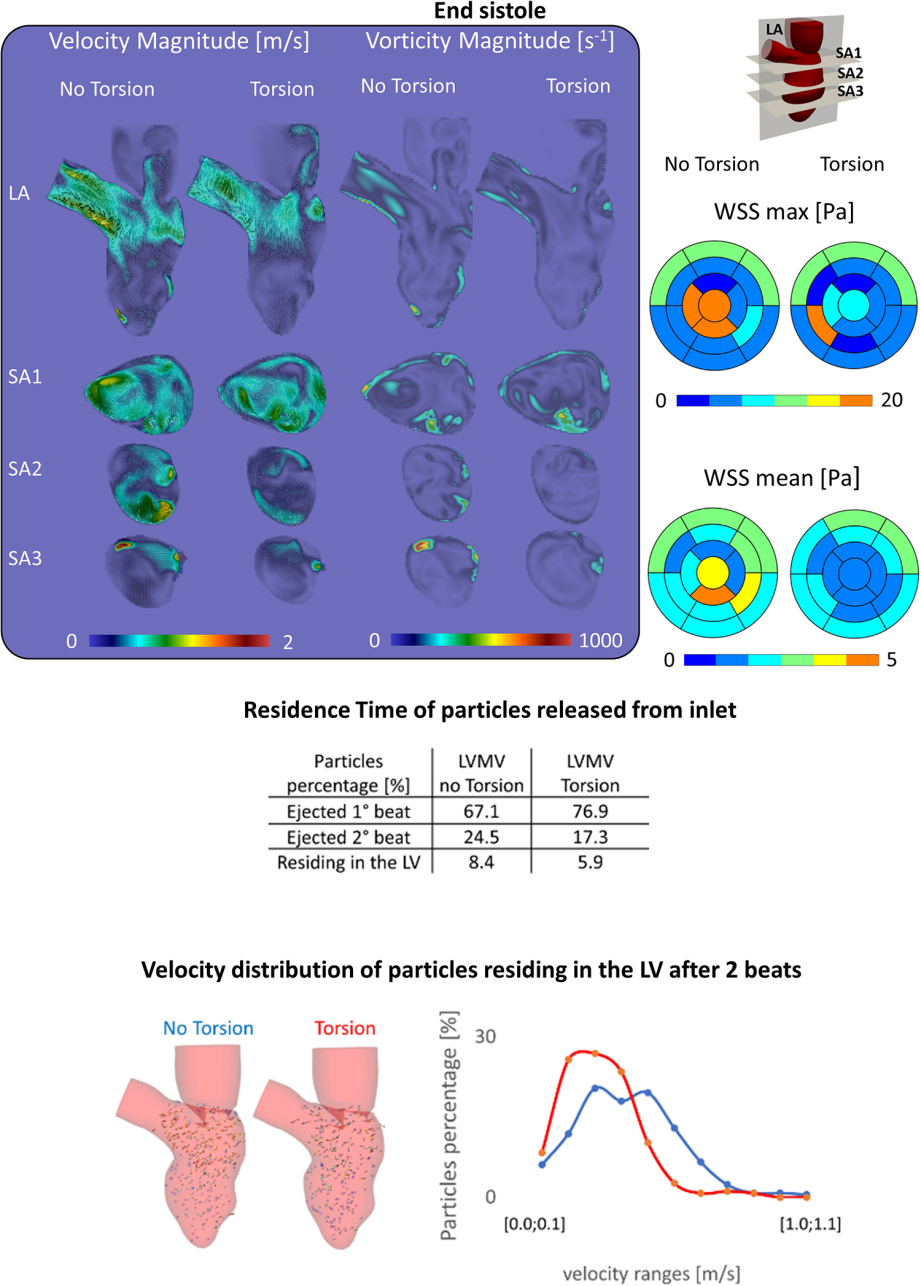
(Top) Velocity, vorticity, wall shear stress contours during end systole show a slight increase of these variables in the case disregarding torsion; (middle) the percentage of particles ejected is higher in the torsion case (+9.8%), nonetheless after two beats the difference decreases below 3%; (Bottom) the case without torsion induces a better motility of the particles residing in the left ventricle.

At the end-systolic configuration (Figure 5, upper panel), the maximum velocity magnitude is around 0.65 m/s and 0.43 m/s, whereas the vorticity magnitude reaches 680 s^−1^ and 520 s^−1^ in the No Torsion and Torsion case, respectively. Omitting torsion leads to a mild increase in velocity and vorticity magnitude in all the investigated planes, but confined to small areas (Supplementary Material Tables S5,S6).

**Figure 6:**
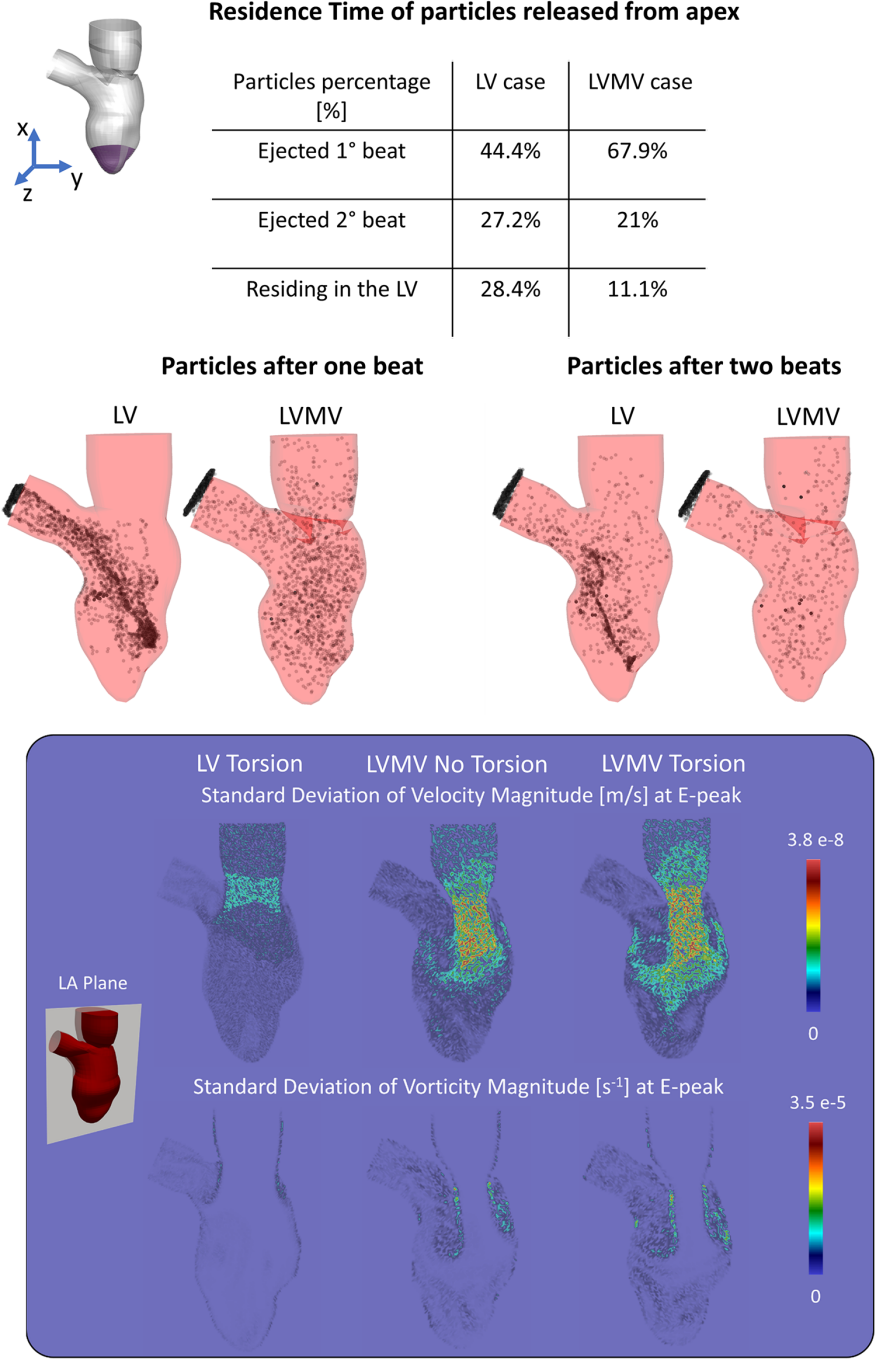
(Top) Residence time of the particles released from the apex show how the mitral valve enhances the apical wash-out. (Bottom) Standard deviation of velocity and vorticity magnitude in the LA plane between the 3rd, 4th, 5th, 6th cycle at the E-peak computed for the case with torsion and without MV (LV Torsion), the case without torsion and with MV (LVMV No Torsion), and the case with torsion and MV (LVMV Torsion).

In a model that incorporates the mitral valve, torsion has a mixed impact on the maximum and mean WSS with a moderate change (increase/decrease) during diastole (changes in the order of 13% and 6%, respectively) and somewhat stronger changes (increase/decrease) at end systole (64% and 25%, respectively). Whether values decreased or increased was dependent on the region considered, as clear from Supplementary Material Tables S7–S10 in the Supplementary Material, where a detailed analysis of maximal and mean WSS in the different ventricular segments for the models with and without the mitral valve and with/without torsion is found.

Energy dissipation (EL) is about 14% higher for physiological torsion (9.7 mJ) over cycle 5 (4.8 mJ) and 6 (4.8 mJ) than when torsion is omitted (8.4 mJ, with 4.1 mJ in cycle 5 and 4.3 mJ in cycle 6).

Figure 5 encompasses the results that were obtained on particle residence times. When particles are released from the inlet, discarding torsion leads to a reduction of the particles ejected within the 1st beat of −9.8% (=76.9%–67.1%) with a consequent increase of both the particles ejected during the 2nd beat (+7.2%) and residing in the LV after two beats (+2.5%) (Supplementary Material Table S1). Within two beats, 91.6% of the particles left the LV without torsion, while this becomes 94.1% when torsion is present. Looking at the velocity of the particles residing after 2 beats, 287 (out of 378) have a velocity lower than 0.5 m/s without torsion; with torsion, this applies to 250 (out of 264) particles.

### Influence of the mitral valve on intraventricular hemodynamics

**Figure 7:**
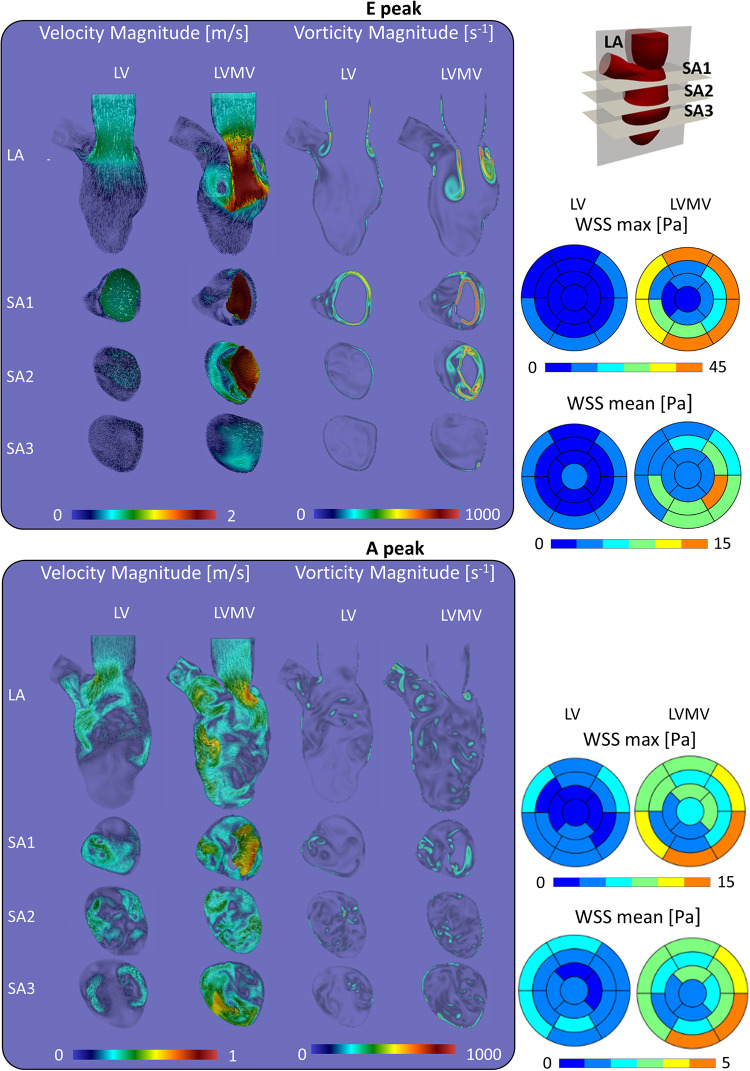
Impact of the mitral valve in the torsion case on the velocity, vorticity and wall shear stress during the diastolic.

At the E-peak (Figure 7), the MV increases the maximum velocity magnitude from 1.0 m/s to 2.0 m/s and extends the areas with velocity beyond 1 m/s by 14%, 3.5%, 23.8%, and 71.5% in the LA, SA1, SA2, SA3 planes, respectively. With the MV, the vortices originate at the tip of the MV leaflets instead of the annulus, resulting in enlarged vortices in the SA1 (the vorticity within 0–250 s^−1^ and beyond 750 s^−1^ increased by 5.3% and by 0.6%, respectively) and in the SA2 plane (the vorticity over 250 s^−1^ increased by 20.8%).

At the A-peak (Figure 7), the MV increases the maximum velocity magnitude from 0.5 m/s to 0.8 m/s, and the areas with velocity beyond 0.25 m/s enlarge by 37.9%, 29.3%, 45.5%, 52% in the LA, SA1, SA2, SA3 planes, respectively. The MV slightly enhances the vorticity above 250 s^−1^ by 1.1%, 4.8%, and 0.9%, in the LA, SA1, and SA3 planes, respectively.

As can be expected from the impact on the velocity field, the presence of the mitral valve increases maximal and mean WSS at all levels in the left ventricle (Supplementary Material Tables S9,S10) with the largest impact for the medial (4–8-fold increase in mean and maximal WSS) and apical segments (2–3-fold increase in mean and maximal WSS).

Focusing on the influence induced by the presence of the mitral valve leaflets in washing out of the LV (Figure 6), we studied clearance of particles released from the apex for a simulation with and without valve (and with torsion applied to both cases). With the valve present, 76.9% and 21% of the particles released from the apical region are ejected during the 1st and 2nd beat, respectively, with the remaining 11.1% of the particles residing in the LV after two beats. In absence of the valve, 44% and 27.2% of the particles were ejected during the 1st and 2nd beat, respectively, with 28.4% residing in the LV.

## Discussion

In this manuscript, we developed a Chimera-based patient-specific model of the LV coupled with a patient-inspired moving MV and showcased the model to assess the impact of torsion and the presence of the mitral leaflets on intraventricular hemodynamics. The open MV was segmented from cine-radial MRI images and leaflet motion was defined using a kinematic model. We tested the Chimera technique because it is particularly suited to combine the large motion of the LV endocardium with the impulsive kinematics of the MV leaflets. The Chimera technique allowed us to overcome the main limitations faced in previous work based on the ALE approach where CFD simulations in a deforming LV with mitral valve limited to one cardiac cycle (17), due to excessively distorted mesh resulting in negative volumes errors, or even to the diastolic phase (30).

Despite the obvious advantages of the Chimera technique over ALE for the given study case, we did experience unexpected technical difficulties while setting up the problem. Initially, the BL meshes of the MV leaflets were separated from the BL mesh of the LV (Figure 1C: separated LVMV): one anterior MV mesh; one posterior MV mesh; one LV mesh, whose generation is described in in (27, 28). With this mesh configuration, however, we experienced sudden vanishing of some component zones during the CFD calculation, regardless of several attempts involving the definition of customized cut-control, the BL thickness, the mesh densities, the temporal interpolation, and smooth connecting angles.

This unforeseen dead zone error forced us to include the MV leaflets directly into the moving LV mesh resulting in limitations for the MV opening angles to preserve the mesh quality. Therefore, despite the claimed advantages of the Chimera technique in terms of handling mesh motion, the software version used in this study should be further optimized. We are aware that the proposed model (with a 2nd order upwind scheme for the convective terms and a 1st order implicit scheme for the temporal discretization) is based on a dissipative numerical scheme that is not ideal to capture the transitional nature of the intraventricular flow field. This numerical scheme was actually imposed by the implementation of the Chimera technique (with further limitations due to the presence of moving boundary conditions) in Fluent software 2019 R3®. The reported limitation is not intrinsic to the Chimera technique as such, but rather dependent on the implementation within software packages, which isn't surprising considering that it is relatively recent (i.e., introduced in Fluent 2018). We tried to minimize the numerical viscosity effect by using reasonably small time steps of 3 ms and a fine grid.

Cycle-to-cycle variation after six cardiac cycles indicates that, even with perfectly repeatable moving (and alternating on-off pressure-wall) boundary conditions, the transitional-to-turbulent flow regime induces differences in the flow field from cycle to cycle. The analysis of the simulated cases highlights that the cycle-to-cycle variation affects more the shape and (marginally) the contours distribution rather than the magnitude of the computed variables. Nonetheless, the effects of the cycle-to-cycle variations might be underestimated in the simulated cases due to the dissipative nature of the numerical schemes. For this reason, cycle-to-cycle variation might be more prominent when using numerical schemes with higher order discretization [as in (12)] and further investigations are required.

Simulating multiple cardiac cycles is a good practice to get rid of the transitional effects, as confirmed by several studies: Long et al. (7), Vasudevan et al. (29), Seo et al. (4), Mangual et al. (31), Chnafa et al. (12) simulated 4, 4, 5, 10, 35 cycles, respectively. Among these, it is worth noticing that only Chnafa et al. (12) reported the cycle-to-cycle variations in the wall shear stress of the diastasis during the 20th and 21st cycles. In the referred case, five cycles were simulated to washout the initial conditions, and the results were reported based on phase-averaging over 30 additional cycles. In the other reported studies, even if multiple cycles were simulated, the analysis is often limited to the last cycle without reporting additional checks about the vanishing of the transient effects (4, 7, 29, 31). More precise recommendations to objectively assess when a CFD calculation has reached a regime state may be helpful to the community. In this context, it is worth remembering that the multi-laboratory study promoted by the FDA to support the use of CFD simulations from a regulatory perspective is at a standstill (32), with CFD-use limited to the design stage and considered appropriate for evaluating relative design changes rather than assessing absolute quantities (33) (ISO 14,708-5). In our case, the RT computation became of interest only after performing the CFD simulations, making it impossible to compute the advection-diffusion-reaction equation using Overset Meshes technique in Fluent 2019 R3. Therefore, the RT computation was based on particle tracking methods. A comparison of the different methodologies to compute residence time (e.g., Eulerian residence time or ERT) in the LV cavity could be of interest as further development.

Overall, we noticed only minor differences in velocity, wall shear stress, and vorticity contours in the simulations with and without torsion (Figures 4 and 5 and Supplementary Material Tables S1–S6). Interestingly, energy dissipation was found about 14% higher in the physiological torsion case compared to the simulation discarding torsion. Nonetheless, given that energy losses are of an order of one thousand of the total cardiac energy (order of a few J) in both simulated cases, the impact of torsion on the energy loss is considered marginal and unlikely of physiological relevance. Moreover, the impact on the energy loss becomes even more marginal considering that the use of low-order dissipative schemes leads to an overestimation of the energy loss. Vasudevan et al. (29) recently investigated the effects of torsional motion on the LV in the fluid dynamics of five healthy human fetal and two healthy adult porcine hearts. The MV geometry was based on detailed anatomical measurements from a database of 10 adult porcine specimens (34) and the opening angles of the leaflets were derived from the three-chamber view of MRI data. Flow and energy dynamics were evaluated varying the torsional degree (0°, 5°, 15°), with and without the mitral valve and the papillary muscles, and under a diseased condition. They found that the impact of ventricular torsion was minor and irrelevant on flow patterns, energy losses, ejection work, and wall shear stress, and the impact on the residence time was not evaluated. In our case, the effects of torsion on velocity and vorticity magnitude were negligible, whereas energy loss increased by 14% when considering torsion. It is hard to assess the physiological significance of this finding; as discussed further, torsion has a meaningful impact on the residence time and particle clearing from the left ventricle, of which the beneficial effects on reducing the risk of blood stasis and thrombus formation may outweigh the energy cost. Indeed, physiological torsion was found to have a favorable effect on removing particles from the LV at the first beat (+9.8%) that reduces up to 2% after two beats. On the other hand, the motility of the particles residing in the LV chamber was reduced by torsion.

To the best of our knowledge, there are no other studies that evaluated the particle residence time in function of the LV torsion, other than our previous study without the MV (28). In that study, however, the mitral valve leaflets were not considered, and given the huge impact of the leaflets on intraventricular hemodynamics (see further), results should not be compared. Mangual et al. (14) injected a virtual tracer inside the LV, of which about 80% is ejected within two beats in the healthy cases (mean ejection fraction and stroke volume equal to 55% and 74 ml, respectively), while this dropped to 20% in the cases with dilated cardiomyopathy (mean ejection fraction and stroke volume equal to 17.8% and 41 ml, respectively). Therefore, the flow after two beats is higher in our case (94.2%). A likely factor leading to this higher value is the high ejection fraction of 67% and stroke volume of 109 ml for this case. Also, the implementation of the MV into the LV mesh limited the opening angle of the valve (to preserve the mesh quality), which may have led to a more energetic incoming jet, generating more swirling flow, and enhancing particle evacuation.

Beyond the study on the torsional effects, we also performed simulations (including torsion) without the MV, well known to have a dramatic impact on intraventricular hemodynamics. Starting from the contours of velocity, vorticity, and WSS, the presence of the mitral valve induced several effects, among which: (i) the formation of the vortical structures at the free edge of the valve instead of the annulus, resulting in a more central position of the vortices with respect to the long axis of the ventricle; (ii) an increased velocity peak of the inlet jet induced by the narrowing of the mitral orifice area; (iii) as a result of the combination of (i) and (ii), the jet and the vortical patterns better penetrate towards the apex, with enhanced local washout, as can be seen by the vorticity and wall shear stress (Figure 7). Our findings about the influence of the valve in the flow field agree with the ones of Bavo et al. (17) and Seo et al. (14). In our case, the apical washout was also assessed by releasing the particles in the apical region and computing their residence time with and without the mitral valve (Figure 6). In this regard, we found out that the valve enhances the direct flow (67.9% vs. 44.4%) and the particles ejected in two beats (88.9% vs. 71.6%). The presence of the valve also has an impact on the faith of particles released from the inlet. In previous work that did not include the mitral valve, physiological torsion decreased the direct flow and the particles ejected within two beats. Conversely, in the current study, torsion enhances the direct flow and the particles ejected within two beats increase. From a clinical perspective, we strongly believe that the residence time and the velocity distribution of the residing particles provide more significant insights to assess the predisposition to stasis rather than velocity, vorticity, or wall shear stress. Torsion does seem to have an impact on these parameters. It may therefore be safe to consider torsion in patient-specific CFD models, especially when these studies target stasis and residence time. In that context, future studies should also evaluate the impact of the Trabeculae Carneae. This will, however, require more advanced mesh handling methods that can cope with small cavities that compress and expand as the ventricle contracts and relaxes.

Our study has some important limitations. The patient-specific LV model has a limited opening angle of the MV, because of the unforeseen dead zone error of the Chimera technique which forced us to include the MV mesh into the LV mesh. This results in a flow field (peak velocity = 2 m/s) more intense than a typical physiological case, which induces instabilities at the tip of the MV at the E-peak. We also believe that the 8 mm slice thickness of our cine-MRI short-axis imaging dataset considerably limited the accuracy of the segmentation of our patient-specific LV model. Therefore, the proposed workflow should be tested on imaging datasets with higher resolution.

The validation of the proposed model could not be performed at the current stage due to limitations of the medical imaging dataset and the Chimera technique (as implemented in Fluent 2019 R3). The low spatial and temporal resolution of the cine-radial MRI imaging dataset prevented the comparison between the patient-inspired kinematic model of the MV and the cine-radial MRI images. Furthermore, the E-peak velocity of 2 m/s imposed by the limitations of the Chimera technique invalidates a direct comparison between the CFD simulations and 4D flow MRI data.

The mitral valve is not sealed during systole resulting in a gap between the MV leaflets during systole. The on-off (pressure-wall) boundary approach was mainly used because in our simulations the flow is driven by the contraction and dilation of the LV chamber, but also because (theoretically) this would have allowed us to avoid regurgitation across the mitral valve during systole given that the volume of our modeled left atrium is fixed. Nonetheless, the minimal motion of the MV leaflets during systole, as indicated by the decrease of the MV orifice area (visible in Figure 2), induces a backflow towards the left ventricle with a peak velocity of 0.4 m/s. Our boundary conditions should be investigated with a new medical imaging dataset that results in a more physiological SV. Furthermore, we could not overcome some important technical difficulties related to the Chimera technique, among which problems with cut-control and insufficient compatibility with the flow solver (e.g., a 2nd order temporal discretization, particles tracking already in Fluent).

The presented model, without turbulence model, can be assumed as a DNS approach able to solve the main large-scale hemodynamic features (such as jets, main vortices, and ejection) scales of the flow field, but it lacks the resolution needed to resolve the smaller flow details. Therefore, much more refined background grids and/or higher order schemes should be further pursued to capture the smaller-scale flow feature of the flow field, transitional flows, and the dissipation of the small scale of the vortices more accurately. Lastly, ventricular torsion was superimposed to the 4D meshes of the LV sac as a global uniform rotation due to the inability to derive this motion component from MRI and CT medical images. A more advanced model that could account for the local variations of the torsional motion might have a relevant impact on our findings.

Finally, only one patient-specific CFD model of the LV has been established. Therefore, caution is warranted to interpret these findings and assess their physiological and clinical relevance. More simulations of physiological and pathological cases are needed to confirm these findings in a sufficient number of cases that is statistically significant. The presented workflow should have the necessary versatility to be applied to both physiological and pathological cases.

## Conclusion

In this study, we presented a Chimera-based patient-specific model of the LV coupled with a patient-inspired kinematic model of the MV. We assessed the impact of torsion in the LV fluid dynamics by simulating multiple cardiac cycles with and without physiological torsion. Our results indicate that torsion has a minimal effect on velocity, vorticity, wall shear stress, and energy loss. With the implementation of the mitral valve, torsion enhanced both the direct flow and (minorly) the particles ejected within two beats, while it reduced the motility of the particles. The MV enhanced the propagation of the inlet jet and promoted both the general and apical washout.

## Data Availability

The original contributions presented in the study are included in the article/**Supplementary Material**, further inquiries can be directed to the corresponding author/s.

## References

[1] World Health Organization. World health organization cardiovascular disease risk charts: revised models to estimate risk in 21 global regions. Lancet Glob. (2019) 7(10):e1332–45. 10.1016/S2214-109X(19)30318-3 PMC7025029 31488387

[2] Morris PD Narracott A Von Tengg-Kobligk H Soto DAS Hsiao S Lungu A Computational fluid dynamics modelling in cardiovascular medicine. Heart. (2016) 102(1):18–28. 10.1136/heartjnl-2015-308044 26512019 PMC4717410

[3] Domenichini F Pedrizzetti G. Asymptotic model of fluid–tissue interaction for mitral valve dynamics. Cardiovasc Eng Technol. (2015) 6(2):95–104. 10.1007/s13239-014-0201-y 26577230

[4] Seo JH Mittal R. Effect of diastolic flow patterns on the function of the left ventricle. Phys Fluids. (2013) 25(11):110801. 10.1063/1.4819067

[5] Mittal R Hee J Vedula V Choi YJ Liu H Huang HH Computational modeling of cardiac hemodynamics: current status and future outlook. J Comput Phys. (2016) 305:1065–82. 10.1016/j.jcp.2015.11.022

[6] Doenst T Spiegel K Reik M Markl M Hennig J Nitzsche S Fluid-dynamic modeling of the human left ventricle: methodology and application to surgical ventricular reconstruction. Ann Thorac Surg. (2009) 87(4):1187–95. 10.1016/j.athoracsur.2009.01.036 19324149

[7] Long Q Merrifield R Xu XY Kilner P Firmin DN Yang GZ. Subject-specific computational simulation of left ventricular flow based on magnetic resonance imaging. Proceedings of the institution of mechanical engineers. H J Med Eng. (2008) 222(4):475–85. 10.1243/09544119JEIM310 18595359

[8] Watanabe H Sugiura S Kafuku H Hisada T. Multiphysics simulation of left ventricular filling dynamics using fluid-structure interaction finite element method. Biophys J. (2004) 87(3):2074–85. 10.1529/biophysj.103.035840 15345582 PMC1304609

[9] Krittian S Janoske U Oertel H Böhlke T. Partitioned fluid–solid coupling for cardiovascular blood flow. Ann Biomed Eng. (2010) 38(4):1426–41. 10.1007/s10439-009-9895-7 20058187

[10] Schenkel T Malve M Reik M Markl M Jung B Oertel H. MRI-based CFD analysis of flow in a human left ventricle: methodology and application to a healthy heart. Ann Biomed Eng. (2009) 37(3):503–15. 10.1007/s10439-008-9627-4 19130229

[11] Chnafa C Mendez S Nicoud F. Image-based large-eddy simulation in a realistic left heart. Comput Fluids. (2014) 94:173–87. 10.1016/j.compfluid.2014.01.030

[12] Chnafa C Mendez S Nicoud F. Image-based simulations show important flow fluctuations in a normal left ventricle: what could be the implications? Ann Biomed Eng. (2016) 44(11):3346–58. 10.1007/s10439-016-1614-6 27073110

[13] Chnafa C Mendez S Moreno R Nicoud F. Using image-based CFD to investigate the intracardiac turbulence. Modeling Simul Appl. (2015) 14(November):113–7. 10.1007/978-3-319-05230-4_4

[14] Seo JH Vedula V Abraham T Lardo AC Dawoud F Luo H Effect of the mitral valve on diastolic flow patterns. Phys Fluids. (2014) 26(12) 1.4904094. 10.1063/1.4904094

[15] Ranganathan N Lam JH Wigle ED Silver MD. Morphology of the human mitral valve. II. the Value Leaflets. Circ. (1970) 41(3):459–67. 10.1161/01.CIR.41.3.459 5415983

[16] Mihalef V Ionasec RI Sharma P Georgescu B Voigt I Suehling M Patient-specific modelling of whole heart anatomy, dynamics and haemodynamics from four-dimensional cardiac CT images. Interface Focus. (2011) 1(3):286–96. 10.1098/rsfs.2010.0036 22670200 PMC3262442

[17] Bavo AM Pouch AM Degroote J Vierendeels J Gorman JH Gorman RC Patient-specific CFD simulation of intraventricular haemodynamics based on 3D ultrasound imaging. Biomed Eng Online. (2016) 15(1):107. 10.1186/s12938-016-0231-9 27612951 PMC5016944

[18] Ma X Gao H Griffith BE Berry C Luo X. Image-based fluid-structure interaction model of the human mitral valve. Comput Fluids. (2013) 71:417–25. 10.1016/j.compfluid.2012.10.025

[19] Su B Zhong L Wang XK Zhang JM Tan RS Allen JC Numerical simulation of patient-specific left ventricular model with both mitral and aortic valves by FSI approach. Comput Methods Prog Bio. (2014) 113(2):474–82. 10.1016/j.cmpb.2013.11.009 24332277

[20] Govindarajan V Mousel J Udaykumar HS Vigmostad SC McPherson DD Kim H Synergy between diastolic mitral valve function and left ventricular flow AIDS in valve closure and blood transport during systole. Sci Rep. (2018) 8(1):1–14. 10.1038/s41598-018-24469-x 29670148 PMC5906696

[21] Gao H Feng L Qi N Berry C Griffith BE Luo X. A coupled mitral valve — left ventricle model with fluid – structure interaction. Med. Eng. Phys. (2017) 47:128–36. 10.1016/j.medengphy.2017.06.042 28751011 PMC6779302

[22] Caballero A Mao W McKay R Sun W. Transapical mitral valve repair with neochordae implantation: fSI analysis of neochordae number and complexity of leaflet prolapse. Int J Numer Method Biomed Eng. (2020) 36(3):1–16. 10.1002/cnm.3297 31833663

[23] Caballero A McKay R Sun W. Computer simulations of transapical mitral valve repair with neochordae implantation: clinical implications. JTCVS Open. (2020) 3(C):27–44. 10.1016/j.xjon.2020.05.010 36003874 PMC9390497

[24] Biffi B Gritti M Grasso A Milano EG Fontana M Alkareef H A workflow for patient-specific fluid – structure interaction analysis of the mitral valve: A proof of concept on a mitral regurgitation case. 2019;(xxxx). 10.1016/j.medengphy.2019.09.020 31653498

[25] Mao W Caballero A McKay R Primiano C Sun W. Fully-coupled fluid-structure interaction simulation of the aortic and mitral valves in a realistic 3D left ventricle model. PLoS One. (2017) 12(9):e0184729. 10.1371/journal.pone.0184729 28886196 PMC5590990

[26] Al-azawy MG Turan A Revell A. An overset mesh approach for valve closure: an LVAD application. Proceedings of the 9th international joint conference on biomedical engineering systems and technologies (BIOSTEC 2016) (2016).1(Biostec):145–51.

[27] Canè F Verhegghe B De Beule M Bertrand PB Van Der Geest RJ Segers P From 4D medical images (CT, MRI, and ultrasound) to 4D structured mesh models of the left ventricular endocardium for patient-specific simulations. Biomed research international (2018). 10.1155/2018/7030718 PMC5817367 29516008

[28] Canè F Selmi M De Santis G Redaelli A Segers P Degroote J. Mixed impact of torsion on LV hemodynamics: a CFD study based on the chimera technique. Comput Biol Med. (2019) 112(July):103363. 10.1016/j.compbiomed.2019.103363 31491610

[29] Vasudevan V Wiputra H Yap CH. Torsional motion of the left ventricle does not affect ventricular fluid dynamics of both foetal and adult hearts. J Biomech. (2019) 96:109357. 10.1016/j.jbiomech.2019.109357 31635847

[30] Bavo AM Pouch AM Degroote J Vierendeels J Gorman JH Gorman RC Patient-specific CFD models for intraventricular flow analysis from 3D ultrasound imaging: comparison of three clinical cases. J Biomech. (2017) 50:144–50. 10.1016/j.jbiomech.2016.11.039 27866678 PMC5191945

[31] Mangual JO Kraigher-krainer E De LA Toncelli L Shah A Solomon S Comparative numerical study on left ventricular fluid dynamics after dilated cardiomyopathy. J Biomech. (2013) 46(10):1611–7. 10.1016/j.jbiomech.2013.04.012 23664278

[32] Stewart SFC Hariharan P Paterson EG Burgreen GW Reddy V Day SW Results of FDA’s first interlaboratory computational study of a nozzle with a sudden contraction and conical diffuser. Cardiovasc Eng Technol. (2013) 4(4):374–91. 10.1007/s13239-013-0166-2

[33] Malinauskas RA Hariharan P Day SW Herbertson LH Buesen M Steinseifer U FDA benchmark medical device flow models for CFD validation. ASAIO Journal. (2017) 63:150–60. 10.1097/MAT.0000000000000499 28114192

[34] Kunzelman KS Cochran RP Verrier ED Eberhart RC. Anatomic basis for mitral valve modelling. J. Heart Valve Dis. (1994) 3(5):491–6 8000582

