# Supplementary material for: A CFD study on the interplay of torsion and vortex guidance by the mitral valve on the left ventricular wash-out making use of overset meshes (Chimera technique)
Source: Front Med Technol. 2022 Dec 22;4:1018058. doi: 10.3389/fmedt.2022.1018058 (PMC9814007; doi:10.3389/fmedt.2022.1018058)
Supplement: Supplementary file 3 [file Datasheet1.docx]

# Supplementary Material

## Tables

| E-peak | velocity [m/s] | [0.0,0.25] | [0.25,0.5] | [0.5,0.75] | [0.75,1.0] | [1.0,1.25] | [1.25,1.5] | [1.5,1.75] | [1.75,2.0] |
| --- | --- | --- | --- | --- | --- | --- | --- | --- | --- |
| LA | No Torsion | 684.6 | 1022.9 | 888.3 | 192.2 | 90.3 | 72.5 | 114.0 | 185.6 |
|  | Torsion | 1055.3 | 705.8 | 850.3 | 182.3 | 91.0 | 71.4 | 131.9 | 162.3 |
| SA1 | No Torsion | 493.7 | 363.7 | 5.2 | 0.0 | 0.0 | 0.0 | 173.7 | 123.0 |
|  | Torsion | 596.8 | 280.9 | 0.2 | 0.0 | 0.0 | 0.0 | 200.5 | 80.7 |
| SA2 | No Torsion | 23.0 | 321.0 | 514.9 | 192.2 | 17.5 | 127.6 | 187.9 | 31.6 |
|  | Torsion | 7.7 | 357.6 | 579.3 | 134.5 | 8.5 | 60.2 | 217.7 | 50.2 |
| SA3 | No Torsion | 148.0 | 1030.3 | 54.9 | 0.0 | 0.0 | 0.0 | 0.0 | 0.0 |
|  | Torsion | 188.0 | 889.6 | 155.7 | 0.0 | 0.0 | 0.0 | 0.0 | 0.0 |

Table 1. Contours extension [mm^2^] of the velocity magnitude at the E-peak.

| E-peak | vorticity [s^-1^] | [0,250] | [250,500] | [500,750] | [750,1000] | [1000,1250] | [1250,1500] |
| --- | --- | --- | --- | --- | --- | --- | --- |
| LA | No Torsion | 3103.8 | 113.6 | 31.8 | 0.2 | 0.0 | 0.9 |
|  | Torsion | 3117.7 | 114.3 | 17.2 | 0.0 | 1.1 | 0.0 |
| SA1 | No Torsion | 1040.9 | 108.5 | 2.3 | 2.0 | 1.8 | 3.6 |
|  | Torsion | 1095.6 | 56.5 | 0.7 | 1.2 | 4.0 | 1.2 |
| SA2 | No Torsion | 1067.2 | 295.9 | 49.8 | 2.8 | 0.0 | 0.0 |
|  | Torsion | 1117.0 | 228.0 | 70.1 | 0.7 | 0.0 | 0.0 |
| SA3 | No Torsion | 1233.3 | 0.0 | 0.0 | 0.0 | 0.0 | 0.0 |
|  | Torsion | 1232.0 | 0.9 | 0.4 | 0.0 | 0.0 | 0.0 |

Table 2. Contours extension [mm^2^] of the vorticity magnitude at the E-peak.

| A-peak | velocity [m/s] | [0.0,0.25] | [0.25,0.5] | [0.5,0.75] |
| --- | --- | --- | --- | --- |
| LA | No Torsion | 862.2 | 2238.5 | 149.6 |
|  | Torsion | 1349.1 | 1801.0 | 100.2 |
| SA1 | No Torsion | 317.2 | 604.3 | 237.7 |
|  | Torsion | 409.6 | 532.7 | 216.9 |
| SA2 | No Torsion | 461.4 | 949.0 | 5.5 |
|  | Torsion | 506.6 | 909.2 | 0.0 |
| SA3 | No Torsion | 391.8 | 840.8 | 0.7 |
|  | Torsion | 495.1 | 638.3 | 99.9 |

Table 3. Contours extension [mm^2^] of the velocity magnitude at the A-peak

| A-peak | vorticity [s^-1^] | [0,250] | [250,500] | [500,750] |
| --- | --- | --- | --- | --- |
| LA | No Torsion | 3176.3 | 73.9 | 0.2 |
|  | Torsion | 3206.1 | 44.2 | 0.0 |
| SA1 | No Torsion | 1093.7 | 65.6 | 0.0 |
|  | Torsion | 1101.0 | 58.2 | 0.0 |
| SA2 | No Torsion | 1405.1 | 10.7 | 0.0 |
|  | Torsion | 1411.7 | 4.0 | 0.0 |
| SA3 | No Torsion | 1218.6 | 14.7 | 0.0 |
|  | Torsion | 1220.7 | 12.6 | 0.0 |

Table 4. Contours extension [mm^2^] of the vorticity magnitude at the A-peak

| ES | velocity [m/s] | [0.0,0.25] | [0.25,0.5] | [0.5,0.75] | [0.75,1.0] | [1.0,1.25] |
| --- | --- | --- | --- | --- | --- | --- |
| LA | No Torsion | 2071.8 | 764.6 | 12.9 | 0.0 | 0.0 |
|  | Torsion | 3074.9 | 0.0 | 0.0 | 0.0 | 0.0 |
| SA1 | No Torsion | 485.8 | 580.0 | 13.8 | 0.0 | 0.0 |
|  | Torsion | 1295.2 | 0.0 | 0.0 | 0.0 | 0.0 |
| SA2 | No Torsion | 623.8 | 258.6 | 20.2 | 0.0 | 0.0 |
|  | Torsion | 998.2 | 0.0 | 0.0 | 0.0 | 0.0 |
| SA3 | No Torsion | 582.4 | 14.0 | 0.7 | 1.0 | 0.2 |
|  | Torsion | 640.1 | 0.0 | 0.0 | 0.0 | 0.0 |

Table 5. Contours extension [mm^2^] of the vorticity magnitude at end systole.

| ES | vorticity [s^-1^] | [0,250] | [250,500] | [500,750] | [750,1000] |
| --- | --- | --- | --- | --- | --- |
| LA | No Torsion | 2931.4 | 15.6 | 1.5 | 0.0 |
|  | Torsion | 3029.2 | 8.9 | 0.0 | 0.0 |
| SA1 | No Torsion | 1111.6 | 50.0 | 2.7 | 0.0 |
|  | Torsion | 1147.1 | 63.6 | 1.0 | 0.0 |
| SA2 | No Torsion | 955.4 | 14.3 | 0.0 | 0.0 |
|  | Torsion | 996.0 | 0.0 | 0.0 | 0.0 |
| SA3 | No Torsion | 587.9 | 4.2 | 4.2 | 2.2 |
|  | Torsion | 627.8 | 2.5 | 0.0 | 0.0 |

Table 6. Contours extension [mm^2^] of the vorticity magnitude at end systole.

| WSS max | No Torsion | Torsion | No Torsion | Torsion | No Torsion | Torsion |
| --- | --- | --- | --- | --- | --- | --- |
| [Pa] | Epeak | | Apeak | | ES | |
| basal | 42.3 | 41.2 | 13.6 | 13 | 12.4 | 12.5 |
| medial | 26.7 | 24.1 | 7.5 | 7.6 | 9.8 | 19.2 |
| apical | 9.1 | 11.9 | 6.9 | 8.6 | 28.2 | 6.9 |
| apex | 6.9 | 6.1 | 6.2 | 5 | 49.1 | 7.3 |

Table 7. Maximum wall shear stress in the No Torsion and Torsion cases reported for the longitudinal sectors of the bull’s eye at the E peak, A peak, and end systole.

| WSS mean | No Torsion | Torsion | No Torsion | Torsion | No Torsion | Torsion |
| --- | --- | --- | --- | --- | --- | --- |
| [Pa] | Epeak | | Apeak | | ES | |
| basal | 6.2 | 6 | 3.7 | 3.5 | 2.5 | 2.4 |
| medial | 8.4 | 8.1 | 2 | 2.1 | 2.2 | 1.6 |
| apical | 3.7 | 3.9 | 2 | 2.2 | 2.7 | 2.5 |
| apex | 3.9 | 3.8 | 1.9 | 1.7 | 4.1 | 1.6 |

Table 8. Mean wall shear stress in the No Torsion and Torsion cases reported for the longitudinal sectors of the bull’s eye at the E peak, A peak, and end systole.

| WSS max | No MV | MV | No MV | MV |
| --- | --- | --- | --- | --- |
| [Pa] | Epeak | | Apeak | |
| basal | 9.3 | 41.2 | 5.9 | 13 |
| medial | 3.2 | 24.1 | 3.8 | 7.6 |
| apical | 4.6 | 11.9 | 2.8 | 8.6 |
| apex | 4.6 | 6.1 | 1.8 | 5 |

Table 9. Maximum wall shear stress in the No MV and MV cases reported for the longitudinal sectors of the bull’s eye at the E peak, A peak, and end systole.

| WSS mean | No MV | MV | No MV | MV |
| --- | --- | --- | --- | --- |
| [Pa] | Epeak | | Apeak | |
| basal | 2.9 | 6 | 1.5 | 3.5 |
| medial | 1.7 | 8.1 | 1.1 | 2.1 |
| apical | 2.8 | 3.9 | 1.1 | 2.2 |
| apex | 3.7 | 3.8 | 0.9 | 1.7 |

Table 10. Mean wall shear stress in the No MV and MV cases reported for the longitudinal sectors of the bull’s eye at the E peak, A peak, and end systole.
